# Effect of differences in extubation timing on postoperative pneumonia following meningioma resection: a retrospective cohort study

**DOI:** 10.1186/s12871-022-01836-w

**Published:** 2022-09-16

**Authors:** Minna Guo, Yan Shi, Jian Gao, Min Yu, Cunming Liu

**Affiliations:** 1 Faculty of Anesthesiology, Changhai Hospital, Naval Medical University, Shanghai, China; 2 Department of Anesthesiology, the Affiliated Wuxi People’s Hospital of Nanjing Medical University, Wuxi, China; 3 School of Public Health, Shanxi Medical University, Taiyuan, China; 4 Department of Anesthesiology and Perioperative Medicine, First Affiliated Hospital of Nanjing Medical University, 210029 Nanjing, China

**Keywords:** Airway extubation, Postoperative pneumonia, Meningioma surgery

## Abstract

**Background:**

This study was designed to examine extubation time and to determine its association with postoperative pneumonia (POP) after meningioma resection.

**Methods:**

We studied extubation time for 598 patients undergoing meningioma resection from January 2016 to December 2020. Extubation time was analysed as a categorical variable and patients were grouped into extubation within 21 minutes, 21–35 minutes and ≥ 35 minutes. Our primary outcome represented the incidence of POP. The association between extubation time and POP was assessed using multivariable logistic regression mixed-effects models which adjusted for confounders previously reported. Propensity score matching (PSM) was also performed at a ratio of 1:1 to minimize potential bias.

**Results:**

Among 598 patients (mean age 56.1 ± 10.7 years, 75.8% female), the mean extubation time was 32.4 minutes. Extubation was performed within 21 minutes (32.4%), 21–35 minutes (31.2%) and ≥ 35 minutes (36.4%), respectively, after surgery. Older patients (mean age 57.8 years) were prone to delayed extubation (≥ 35 min) in the operating room, and more inclined to perioperative fluid infusion. When extubation time was analysed as a continuous variable, there was a U-shaped relation of extubation time with POP (*P* for nonlinearity = 0.044). After adjustment for confounders, extubation ≥35 minutes was associated with POP (odds ratio [OR], 2.73 95% confidence interval [CI], 1.36 ~ 5.47). Additionally, the results after PSM were consistent with those before matching.

**Conclusions:**

Delayed extubation after meningioma resection is associated with increased pneumonia incidence. Therefore, extubation should be performed as early as safely possible in the operation room.

**Supplementary Information:**

The online version contains supplementary material available at 10.1186/s12871-022-01836-w.

## Background

Meningioma is the most common primary brain tumor, accounting for about 30% of central nervous system (CNS) tumors in adults [1]. For tumors that are growing or causing symptomatology, maximal surgical resection under general anaesthesia with intubation remains the standard of care for therapeutic management of meningioma [2]. The particularity of neurosurgery is that an altered state of consciousness impairs the respiratory, which in turn may reduce brain functions [3]. Thus, the incidence of postoperative pneumonia (POP) is high after craniotomy.

POP, one of the most common postoperative pulmonary complications (PPCs) after surgery, not only prolongs the hospital stay, but even increases the risk of other severe complications and postoperative mortality [4, 5]. Previous studies have shown that early extubation is associated with reduced PPCs and hospital stay after major surgery, but they mostly compared the prognosis of patients with extubation in the intensive care unit (ICU) and those patients with extubation in operating room [6–11]. To date, there are no studies regarding the optimal timing of extubation of ventilated patients after craniotomy in the operating room. And few studies explored the relationship between extubation time and POP in the neurosurgery subgroup. But it’s worth noting that the control of extubation time in the operative room is more clinically instructive for anesthesiologists. And early recovery of patients’ breathing and consciousness is conducive to neurosurgeons’ neurological evaluation. Thus, we aimed to explore the association between extubation time and POP after meningioma resection in the operation room and provided it for clinical practice as a reference.

## Methods

This retrospective study was approved by the Institutional Review Board of the First Affiliated Hospital of Nanjing Medical University (IRB: 2021-SR-047), and the requirement for written informed consent was waived by the institutional review board. The Strengthening the Reporting of Observational Studies in Epidemiology (STROBE) checklist for cohort studies was referenced when we performed this study. All experimental procedures were performed in accordance with relevant guidelines and regulations (declarations of Helsinki).

### Patient population

We determined the sample size from a similar study of previous non-cardiac surgery [6, 11–13]. And we retrospectively analyzed 820 patients who underwent meningioma resection at the First Affiliated Hospital of Nanjing Medical University from January 2016 to December 2020. We included patients aged >18y with the American Society of Anesthesiologists (ASA) physical status I-III. The exclusion criteria were as follows: (1) severe cardiac /lung /liver or renal insufficiency (The definition is in the Additional file 1. eTable 1); (2) preoperative lung infection or other organ infections; (3) incomplete clinical information; (4) emergency; (5) failure to reach extubation criteria in the operating room after surgery. In addition, to reduce unnecessary medical expenses, patients undergoing elective meningioma surgery in our hospital were usually extubated in the operative room, so we excluded the patients who unplanned admission to ICU.

### Data collection

Data were obtained from the Hospital Information System, which could be listed as follows: (1) preoperative-related variables including sex, age (>18y), body mass index (BMI), preoperative co-morbidities (hypertension, diabetes, coronary heart disease (CAD), history of smoking, history of epilepsy, history of tumor, previous meningioma resection surgery, tumor size (maximum diameter), the World Health Organization (WHO) class of meningioma (I-IV); (2) intraoperative-related variables including ASA status (I-III), surgery duration, total volume of input fluid, total volume of blood loss, tracheal extubation time, time of post-anesthesia care unit (PACU) stay; (3) postoperative-related variables including ICU admission (yes/no), time of ICU stay, postoperative complications (POP, intracranial infection, coma, brain edema, intracranial hematoma, deep venous thrombosis (DVT)), post-surgical hospital stay, readmission rate. Among them, extubation time was defined as the time (in minutes) from the end of surgery to the time of extubation in the operating room.

### Endpoints, exposures, and confounders

The primary outcome of this retrospective analysis was the incidence of POP, defined as a respiratory infection requiring antibiotic treatment and meeting at least one of the following criteria: new or changed sputum, new or changed lung opacities on a clinically indicated chest radiograph, temperature > 38.3 °C, leucocyte count > 12,000 μL^− 1^ [14, 15].

The primary exposure factor in our study was tracheal extubation time which is defined as the end of surgery to the removal of a tracheal tube. The decision regarding tracheal extubation time comprised a response to verbal commands, a positive gag reflex, tidal volumes > 6 mL/kg, respiratory rate < 20 times/min, oxygen saturation > 96% while breathing spontaneously (FiO_2_ ≤ 50%), normocarbia judged by end-tidal carbon dioxide, reversal of neuromuscular blockade judged by peripheral nerve stimulator or clinical assessment, core body temperature between 36.5 °C and 37.5 °C, and hemodynamics was stable without the support of vasoactive drugs.

After considering previous definitions of delayed awakening (20 min, 30 min) [16, 17], preliminary results of data analysis (Additional file 2. eFigure 1), clinical significance and balance of sample size between groups, our research team divided the data into 3 groups (less than 21 min, 21-35 min and more than 35 min) to explore (1) whether patients with shorter extubation time (less than 21 min) had a lower risk of POP (2) or whether patients with delayed extubation time had a high risk of POP (more than 35 min).

As per previous reports, age, male, low BMI, secondary surgery, preoperative combined cardiovascular disease, and history of smoking constitute preoperative risk factors of POP. Thus, we considered these factors as potential confounders in the analysis [4, 18, 19].

### Statistical analysis

Patients with incomplete data were excluded. Continuous data were expressed as mean and standard deviation (SD) or median and interquartile range, as appropriate. Categorical variables were expressed as frequencies (percentages). The difference of normally distributed data was analyzed using Analysis of Variance (ANOVA), while data with skewed distribution were compared by Kruskal-Wallis test. Also, the χ^2^ test and Fisher’s exact test were employed to analyze categorical variables.

Besides, to explore confounding, we entered covariates into a logistic model in the basic model or eliminated the covariates in the complete model one by one and compared the regression coefficients. In this study, the logistic models were adjusted for age, sex, BMI, previous meningioma resection surgery, size of tumor, preoperative combined cardiovascular disease, history of smoking, ASA score, and WHO classification.

A generalized additive model was used to assess the nonlinear relationship between extubation time and incident POP. We conducted restricted cubic spline models to develop OR curves to examine the possible nonlinear time-response associations between extubation and POP.

Furthermore, to minimize the potential bias of treatment allocation and confounding, we generated a propensity score by logistic regression to balance patients’ preoperative baseline. А 1:1 nеаrеѕt nеіghbоr mаtсhіng аlgоrіthm wаѕ аррlіеd uѕіng а caliper width of 0.01. The following variables were selected to generate the propensity score: age, gender, BMI, hypertension, diabetes, CAD, history of smoking, previous meningioma resection surgery, tumor size, and WHO classification. Afterwards, a standardized mean difference (SMD) was used to examine the PSM degree, and a threshold of less than 0.1 was considered acceptable.

A statistically significant difference was interpreted as a *P*-value of < 0.05. All the analyses were performed with the SPSS 25.0 software (SPSS, Inc., Chicago, IL, USA) and the statistical software packages R (http://www.R-project.org, The R Foundation) and Free Statistics software version 1.3.

## Results

### Study population recruitment summary

**Fig. 1:**
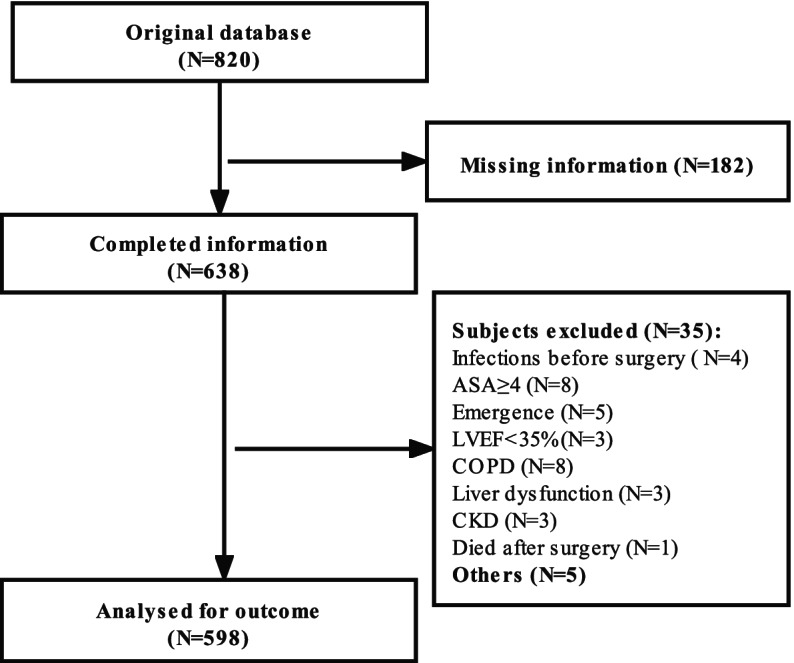
Study population recruitment summary. Abbreviations: ASA, American Society of Anesthesiologists; COPD, chronic obstructive pulmonary disease; CKD, chronic kidney disease; LVEF, left ventricular ejection fraction

From January 2016 to December 2020, a total of 820 patients underwent meningioma resection. Of them, 182 were excluded for missing clinical information, 35 for ineligible criteria, 5 for other causes, and finally 598 were taken into analysis (Fig. 1).

### Demographic and clinical characteristics

Table 1 showed patients in this study and their perioperative characteristics. From January 2016 to December 2020, the mean age at symptom onset was 56.1 ± SD 10.7 years, and there were 453 female and 145 male patients. The patients in the delayed extubation cohort (≥ 35 min) were older (mean age 57.8 years), and harboured a higher incidence of POP (12.8%) than the other two groups. Although there was no significant difference in other preoperative baselines such as tumor size, WHO score, ASA score, surgery or anaesthesia duration time between the two groups, the patients in the delayed extubation cohort (≥ 35 min) had more fluid infusion (median volume 2100 vs. 2000 ml, *P* = 0.050).

**Table 1:** Baseline characteristics of meningioma patients by categories of extubation time, 2016–2020

	Total (*n* = 598)	< 21 min (*n* = 194)	21-35 min (*n* = 186)	≥ 35 min (*n* = 218)	*P*-value
***Preoperative baseline***					
Age (years)	56.1 ± 10.7	54.3 ± 11.1	56.0 ± 11.2	57.8 ± 9.6	0.004
Sex (%)					0.631
Male	145 (24.2)	47 (24.2)	41 (22)	57 (26.1)	
Female	453 (75.8)	147 (75.8)	145 (78)	161 (73.9)	
ASA (%)					0.102
I	51 (8.5)	24 (12.4)	15 (8.1)	12 (5.5)	
II	507 (84.8)	161 (83)	157 (84.4)	189 (86.7)	
III	40 (6.7)	9 (4.6)	14 (7.5)	17 (7.8)	
BMI (kg/m2)	24.4 ± 3.3	24.7 ± 3.3	24.3 ± 3.5	24.1 ± 3.2	0.202
Co-morbidities (%)					
History of smoking	15 (2.5)	2 (1)	9 (4.8)	4 (1.8)	
Hypertension	227 (38.0)	72 (37.1)	67 (36)	88 (40.4)	
Diabetes	65 (10.9)	19 (9.8)	23 (12.4)	23 (10.6)	
CAD	18 (3.0)	5 (2.6)	7 (3.8)	6 (2.8)	
History of epilepsy	23 (3.9)	7 (3.6)	9 (4.8)	7 (3.2)	
Size of maximum tumor diameter (mm)	32.8 ± 17.8	32.6 ± 20.2	33.8 ± 16.3	32.0 ± 16.9	0.677
WHO grade of meningiomas (%)					0.799
I	515 (86.1)	172 (88.7)	159 (85.5)	184 (84.4)	
II	50 (8.4)	14 (7.2)	14 (7.5)	22 (10.1)	
III	13 (2.2)	3 (1.5)	4 (2.2)	6 (2.8)	
IV	3 (0.5)	0 (0)	2 (1.1)	1 (0.5)	
***Intraoperative***					
Surgery duration (min)	160.0 (116.0, 220.0)	157.5 (112.8, 220.0)	160.0 (112.8, 215.0)	165.5 (120.0, 225.0)	0.616
Anaesthesia duration (min)	190.0 (145.0, 255.0)	180.0 (145.0, 255.0)	190.0 (143.0, 250.0)	200.0 (146.2, 255.0)	0.641
Total of infusion (ml)	2000.0 (1600.0, 2596.0)	2000.0 (1500.0, 2600.0)	2000.0 (1600.0, 2500.0)	2100.0 (1600.0, 2600.0)	0.050
Total of loss (ml)	1000.0 (600.0, 1500.0)	1000.0 (550.0, 1500.0)	900.0 (550.0, 1450.0)	1100.0 (687.5, 1500.0)	0.287
***Postoperative***					
Extubation time (min)	32.4 ± 20.2	14.2 ± 5.1	27.8 ± 3.3	52.5 ± 19.3	< 0.001
PACU stay (min)	67.5 ± 28.5	47.6 ± 14.8	61.7 ± 16.5	89.9 ± 30.2	< 0.001
ICU admission (%)	19 (3.2)	8 (4.1)	5 (2.7)	6 (2.8)	0.450
POP	50 (8.4)	14 (7.2)	8 (4.3)	28 (12.8)	0.005
Postoperative complication					0.786
Coma	7 (1.2)	2 (1)	3 (1.6)	2 (0.9)	
Hemorrhage	13 (2.2)	4 (2.1)	3 (1.6)	6 (2.8)	
Intracranial infections	47 (7.9)	16 (8.3)	17 (9.1)	14 (6.4)	
DVT	46 (7.7)	10 (5.2)	12 (6.5)	24 (11)	
Brain edema	15 (2.5)	4 (2.1)	7 (3.8)	4 (1.8)	
Hospital Stay (days)	10.9 ± 4.7	10.6 ± 4.7	11.1 ± 4.7	11.0 ± 4.8	0.519
Readmission rate (%)	8 (1.3)	2 (1)	4 (2.2)	2 (0.9)	0.607
Second surgery (%)	6 (1.0)	3 (1.5)	0 (0)	3 (1.4)	0.121

Normally distributed data are presented as mean (SD), which were analyzed ANOVA; non-normal data are presented as median (range), which were analyzed using Kruskal–Wails test; categorical variables are presented as count (%), which were analyzed using the χ2 test and Fisher’s exact test

*Abbreviations*: *ASA* American Society Anesthesiologists, *BMI* Body mass index, *CAD* Coronary artery disease, *DVT* Deep venous thrombosis, *ICU* Intensive care unit, *PACU* Post-Anesthesia Care Unit, *POP* Postoperative pneumonia, *WHO* The World Health Organization

### Association between extubation time and POP

Results of the univariate analysis are summarized in Table 2, ASA score, history of CAD, WHO score, blood loss, surgery or anaesthesia duration time and extubation time were associated with POP. We therefore adjusted for these confounding factors and other confounders previously reported by the multivariable logistic regression model.

**Table 2:** Univariate logistic regression analysis of covariates for POP

	OR (95%CI)	*P* value
Age	1.01 (0.98 ~ 1.04)	0.552
Gender		
Male	1	
Female	0.73 (0.38 ~ 1.37)	0.323
ASA score		
I	1	
II	2.1 (0.49 ~ 8.95)	0.316
III	6.12 (1.22 ~ 30.7)	0.028
BMI	1.04 (0.95 ~ 1.13)	0.371
History of smoking		
No	1	
Yes	2.85 (0.78 ~ 10.46)	0.114
Hypertension		
No		
Yes	1.57 (0.88 ~ 2.81)	0.129
Diabetes		
No	1	
Yes	0.69 (0.24 ~ 2)	0.498
CAD		
No	1	
Yes	3.32 (1.05 ~ 10.49)	0.041
Size of tumor	1.01 (1 ~ 1.03)	0.057
WHO score		
I	1	
II	0.7 (0.21 ~ 2.35)	0.564
III	2 (0.43 ~ 9.3)	0.379
IV	21.95 (1.95 ~ 247.06)	0.012
Surgery duration	1.01 (1 ~ 1.01)	< 0.001
Blood loss	1.00 (1.00 ~ 1.00)	0.005
Anaesthesia duration	1.01 (1 ~ 1.01)	< 0.001
Extubation Time	1.02 (1.01 ~ 1.03)	0.001
Extubation Time		
< 35 min	1	
≥ 35 min	2.59 (1.34 ~ 5.02)	0.005

*Abbreviations*: *ASA* American Society Anesthesiologists, *BMI* Body mass index, *CI* Confidence interval, *ICU* Intensive care unit, *OR* Odds ratio, *WHO* The World Health Organization

**Fig. 2:**
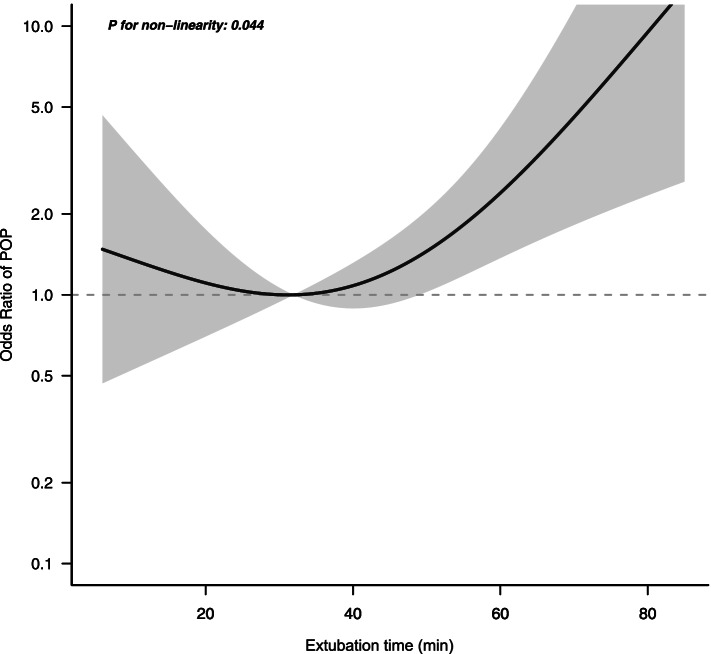
Associations between extubation time and POP. Adjusted for age, gender, BMI, ASA score, surgery duration, history of smoking, size of tumor and WHO score

Figure 2 depicted a generalized additive model that predicted POP based on extubation time, adjusting for age, gender, BMI, ASA, surgery duration, history of smoking, blood loss, previous meningioma resection surgery, preoperative combined CAD, size of tumor and WHO classification. Overall, the association between extubation and POP followed a U-shape. The relationship between extraction time and POP is shown in fig. 2: the slope of the line decreases and then increases with increasing extraction time, with the lowest point of the line at 30–40 minutes. Adjusted splines simulated an area of inflection around 35 minutes when the risk of developing POP began to increase (Supplementary Table 2 in the supplement). The maximum area under the curve increased when patients were divided into early or delayed extubation groups using 35 minutes. Delayed extubation was therefore defined as extubation occurring more than 35 minutes after surgery. Also, patients who developed POP showed a longer extubation time than that of patients without POP (41.6 ± 25.8 vs 31.5 ± 19.4 min, *P* <  0.001) (Supplementary Fig. 2).

Table 3 showed ORs and 95% CIs for risk of incident POP. In the crude model, there was a 1.40-fold increased risk of POP compared to a shorter extubation time cohort (< 35 min) (OR: 2.40, 1.34 to 4.31, *P* = 0.003). In multivariable logistic regression analyses, after adjusting for age, gender and BMI, the OR represented 2.24 (1.22 ~ 4.09). After adjustment for intraoperative and preoperative relevant covariates, we observed that the ORs of delayed extubation cohort (≥ 35 min) were consistently significant in all four models (2.60 (1.32 ~ 5.11), 2.46 (1.31 ~ 4.62), respectively). After adjustment for all covariates in table 2, the risk was increased by 1.73 times in the incidence of POP in the delayed extubation cohort (≥ 35 min) compared to shorter extubation time cohort (< 35 min). (OR:2.73, 1.36 to 5.47, *P* = 0.005). In PSM analysis, with matching age, gender, BMI, hypertension, diabetes, CAD, history of smoking, previous meningioma resection surgery, tumor size and WHO classification, the risk was increased by 1.81 times in the incidence of POP in the delayed extubation cohort (≥ 35 min) compared to shorter extubation time cohort (< 35 min) (OR: 2.81, 1.42 to 5.56, *P* = 0.003). After PSM adjustment, the association remained significant and was not affected by potential confounders (OR: 2.54, 1.16 to 5.53, *P* = 0.019).

**Table 3:** Associations between extubation time and POP in the crude analysis, multivariable analysis, and propensity-score analyses

	Extubation time ≥ 35 min	*P*-value
Crude analysis- OR (95% CI)	2.40 (1.34 ~ 4.31)	0.003
Multivariable analysis- OR (95% CI)		
Adjust I	2.24 (1.22 ~ 4.09)	0.009
Adjust II	2.60 (1.32 ~ 5.11)	0.006
Adjust III	2.46 (1.31 ~ 4.62)	0.005
Adjust IV	2.73 (1.36 ~ 5.47)	0.005
PSM analysis- OR (95% CI)		
With matching ^a^	2.81 (1.42 ~ 5.56)	0.003
Adjusted for propensity score ^b^	2.54 (1.16 ~ 5.53)	0.019

Adjust I model adjusted for age, gender and BMI; adjust II model adjusted for adjust I + ASA score, blood loss and surgery duration; adjust III model adjusteds for adjust I + previous meningioma resection surgery, preoperative combined cardiovascular disease, history of smoking, size of tumor and WHO classification, adjust IV model adjusts for adjust I + ASA, surgery duration, history of smoking, blood loss, previous meningioma resection surgery, preoperative combined cardiovascular disease, size of tumor and WHO classification

*Abbreviations*: *ASA* American Society Anesthesiologists, *BMI* Body mass index, *CI* Confidence interval, *OR* Odds ratio, *PSM* Propensity score matching, *WHO* The World Health Organization

PSM analysis matched age, gender, BMI, hypertension, diabetes, coronary artery disease, history of smoking, previous meningioma resection surgery, tumor size and WHO classification. ^a^ Shown is the OR from a univariate logistic regression model with matching according to the propensity score. ^b^ Shown is the OR from a multivariable logistic regression model, with additional adjustment for the propensity score

## Discussion

Our data demonstrated that extubation time was associated with POP, in addition to well-known prognostic factors such as age, ASA score, history of CAD, WHO score, surgery duration and anaesthesia duration. Interestingly, anesthesiologists wait longer to extubate the elderly and patients who require a higher fluid infusion. While it was not surprising that patients with delayed extubation had a worse outcome, we discovered that early extubation within 35 minutes in the operating room, rather than in the ICU, had an independent effect.

Consistent with our results, previous studies have shown that early extubation is associated with reduced PPCs and shorter hospital stay after major surgery, but few have focused on the neurosurgical subgroup [6–11]. However, early recovery of patients’ breathing and consciousness is conducive to neurosurgeons’ neurological evaluation, especially in the “rapid awaking strategy” [3]. Our study extended these findings to explore a safe and optimal extubation time in neurosurgical patients.

Fandler-Höfler S et al. conducted a retrospective cohort study in the neurointensive care unit or stroke unit stay (*n* = 447) and described longer ventilation time was associated with a higher rate of pneumonia during neurointensive care unit or stroke unit stay (early (< 6 h)/ delayed (6 ~ 24 h)/ late (> 24 h) extubation: 9.6%/20.6%/27.7%, *P* <  0.01) [11]. In Nikoubashman O′ cohort, prolonged ventilation (> 24 h) was associated with POP during hospitalization and unfavorable functional outcome and death at follow-up (*P* ≤ 0.001) [10]. Different from previous studies, our design setting focused on the patients met immediate extubation criteria within the operating room. Interestingly, the same phenomenon also can be found in our study, delayed extubation (> 35 min in the operation room) was significantly associated with POP in patients after meningioma resection.

The reasons underlying the association between intubation duration and POP may be relative to pulmonary dysfunction. On the one hand, the particularity of neurosurgery is that lung dysfunction can be caused by damage to functional brain areas, neurological paralysis, or enteral malnutrition, et al. [20], which in turn may reduce brain function [3]. General anaesthesia with intubation, on the other hand, disrupts physiological lung function due to changes in chest wall mechanics and diaphragm relaxation, causing atelectasis, gas exchange disruption, and ventilation-perfusion mismatch [21]. Additionally, barotrauma, volutrauma, atelectotrauma, and biotrauma are partly responsible for ventilator-induced lung injury [22].

It is noteworthy that patients with delayed extubation had more intraoperative fluid infusion, so the reason of delayed extubation may be related to pulmonary and cerebral edema, especially in patients with massive blood loss [20, 23]. Considering the administration of diuretics and the risk of bleeding during major neurosurgery, anesthesiologists need to balance the basic fluid requirement. Because fluid overload could result in pulmonary edema, cerebral edema or acute kidney injury. Careful selection of the type of infusion fluid (crystalloid or colloid therapy), and the use of goal-directed fluid therapy (GDFT) may help to manage fluid balance in neurosurgical patients who are dehydrated due to administration of diuretics [24–26]. Controlling fluid balance to avoid pulmonary and cerebral edema may allow patients to be extubated sooner after surgery.

Patients in the delayed extubation cohort were older than those in the cohort with a shorter extubation time. Our result was akin to many other findings. Most of the previous research had examined that older age could be the independent factor for delayed extubation, which was in turn associated with worse outcomes [27, 28]. Elderly patients have increased sensitivity toward general anesthetics, and slow return of consciousness due to progressive decline in CNS function [29]. Not to mention, craniotomy itself can cause CNS dysfunction [20]. But with the progress of medical technology and increasing human life expectancy, an increasing number of meningiomas has been identified in elderly patient. Thus, anesthesiologists need to weigh against benefits of early extubation in elderly patients.

The limitation of this study is that it was a single-center, retrospective study. We collected as many sample sizes as possible, and rigorous data analysis was performed to demonstrate the relationship between airway extubation time and POP. Furthermore, the incidence of these complications depends largely on the difficulty of treatment. Easily accessible meningiomas are far more different from complicated skull-base ones. The former shared more limited incisions, shorter surgery time duration, as well as fewer blood transfusions [19, 20]. And the post-operative intensive care of skull-base meningiomas resection may be reserved for intubation and mechanical ventilation, resulting in a significant increase in POP. For this reason, we excluded patients who entered the ICU after surgery. The highlight of our study lies in the inclusion of patients who could be extubated in the operating room and met the extubation criteria, providing a reference for anesthesiologists working in the operating room.

## Conclusion

In conclusion, delayed extubation after meningioma resection is associated with increased pneumonia incidence. Thus, appropriate patients should be extubated as early as possible in the operating room to decrease the incidence of POP. By doing so, it may help to reduce the use of ICU and specialized staff without altering other clinical postoperative complications and hospital length of stay.

## Supplementary Information

**Additional file 1.**

**Additional file 2.**

## Data Availability

The data are not available for public access because of patient privacy concerns, but are available from the corresponding author on reasonable request.

## Abbreviations

ASA: American Society of Anesthesiologists
ANOVA: Analysis of Variance
BMI: Body mass index
CAD: Coronary heart disease
CNS: Central nervous system
CI: Confidence interval
DVT: Deep venous thrombosis
GDFT: Goal-directed fluid therapy
ICU: Intensive care unit
OR: Odds ratio
POP: Postoperative pneumonia
PSM: Propensity score matching
PPCs: Postoperative pulmonary complications
PACU: Post-anesthesia care unit
STROBE: Strengthening the Reporting of Observational Studies in Epidemiology
SMD: Standardized mean difference
WHO: World Health Organization
