# Supplementary material for: Effect of differences in extubation timing on postoperative pneumonia following meningioma resection: a retrospective cohort study
Source: BMC Anesthesiol. 2022 Sep 16;22:296. doi: 10.1186/s12871-022-01836-w (PMC9479244; doi:10.1186/s12871-022-01836-w)
Supplement: Supplementary file 1 — Additional file 1. [file 12871_2022_1836_MOESM1_ESM.docx]

**Supplementary information**

**Supplementary Table 1. T****he definition of preoperative comorbidities**

| ***Disease*** | ***Definition*** | ***Clinical information*** |
| --- | --- | --- |
| Severe cardiac insufficiency | Active heart disease, recent myocardial infarction (≤ 1 month before surgery), heart failure, significant arrhythmia, severe valvular disease | Medical records, ECG, echocardiography, Biomarkers (BNP, N-pro-BNP). (and if had, Preoperative Coronary Angiography) |
| Severe lung insufficiency | Preoperative lung infection | Medical records, Preoperative chest X-ray |
| Severe liver insufficiency | Child-Pugh C score | Medical records, serum bilirubin, prothrombin time, serum albumin |
| Severe renal insufficiency | Chronic kidney disease more than 3 stage | Whole blood creatinine |

**Supplementary Table 2. Estimated break point of extubation time**

|  | OR | *P* value |
| --- | --- | --- |
| Estimated break point | 34.69 (34.09, 35.291) |  |
| Slope 1 | 0.894 (0.818~0.977) | 0.0134 |
| Slope 2 | 1.033 (0.995~1.072) | 0.0904 |
| Likelihood Ratio test | - | 0.01 |

Adjusted for age, gender, BMI, ASA score, surgery duration, history of smoking, size of tumor and WHO score.
