# Supplementary material for: Effect of differences in extubation timing on postoperative pneumonia following meningioma resection: a retrospective cohort study
Source: BMC Anesthesiol. 2022 Sep 16;22:296. doi: 10.1186/s12871-022-01836-w (PMC9479244; doi:10.1186/s12871-022-01836-w)
Supplement: Supplementary file 2 — Additional file 2. [file 12871_2022_1836_MOESM2_ESM.docx]

**Supplementary Figure 1. Associations between extubation time and POP**


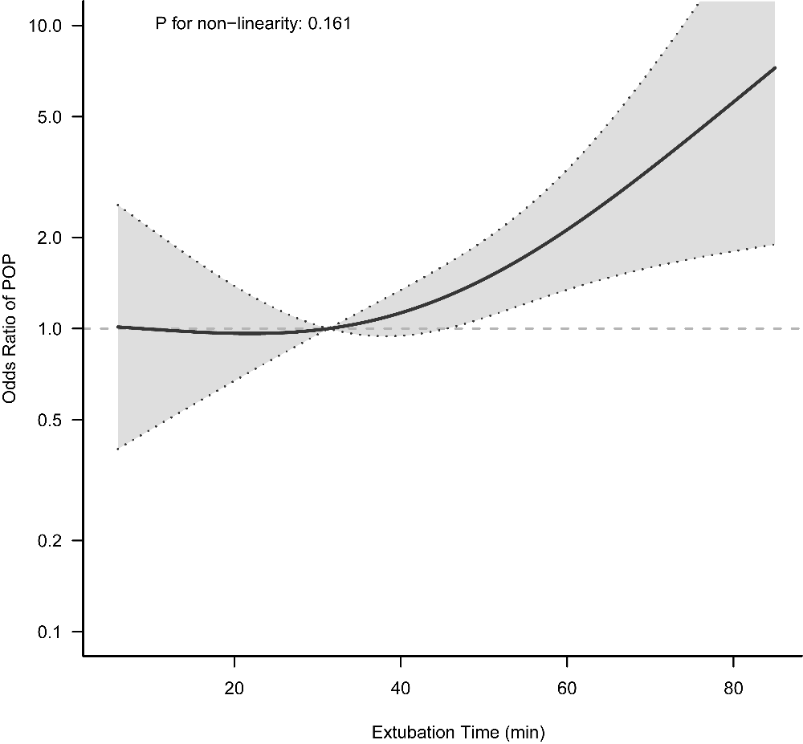


Non-linear relationship of extubation time and POP without adjusted for age, gender, BMI, ASA score, surgery duration, history of smoking, size of tumor and WHO score.

**Supplementary Figure 2.** **Extubation time in the pneumonia and non-pneumonia group**


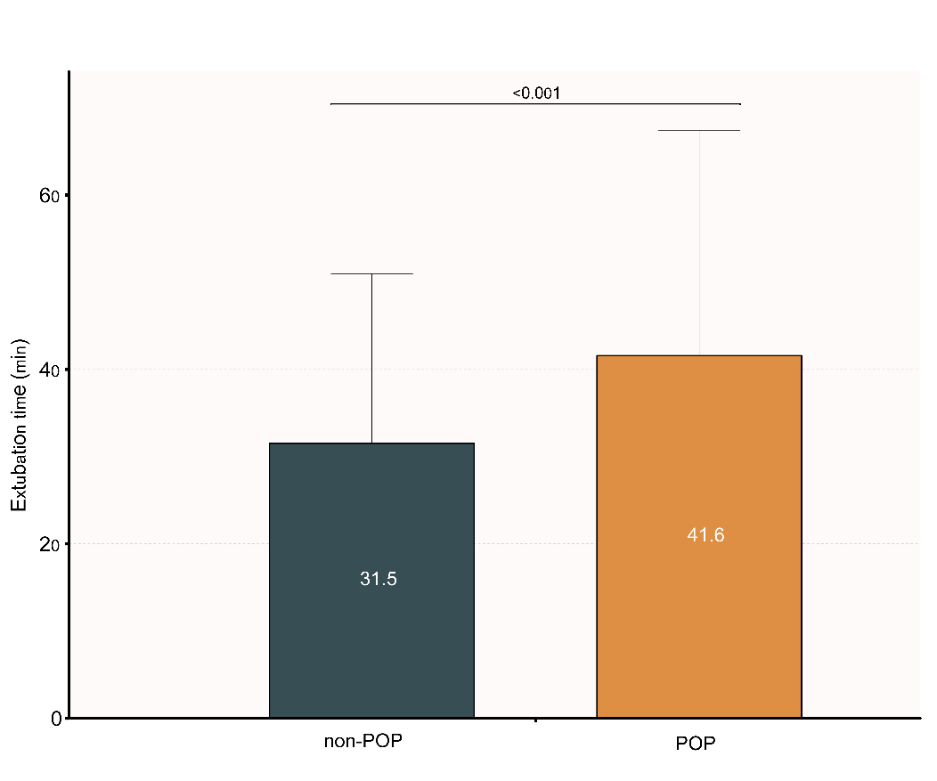


Histogram comparing the mean time to airway extubation (measured from surgery). The mean time to extubation significantly longer in the pneumonia group (41.5min *vs* 31.5min, *P* < 0.001, t-test).

**Supplementary Figure 3. The SMD of the degree of PSM**


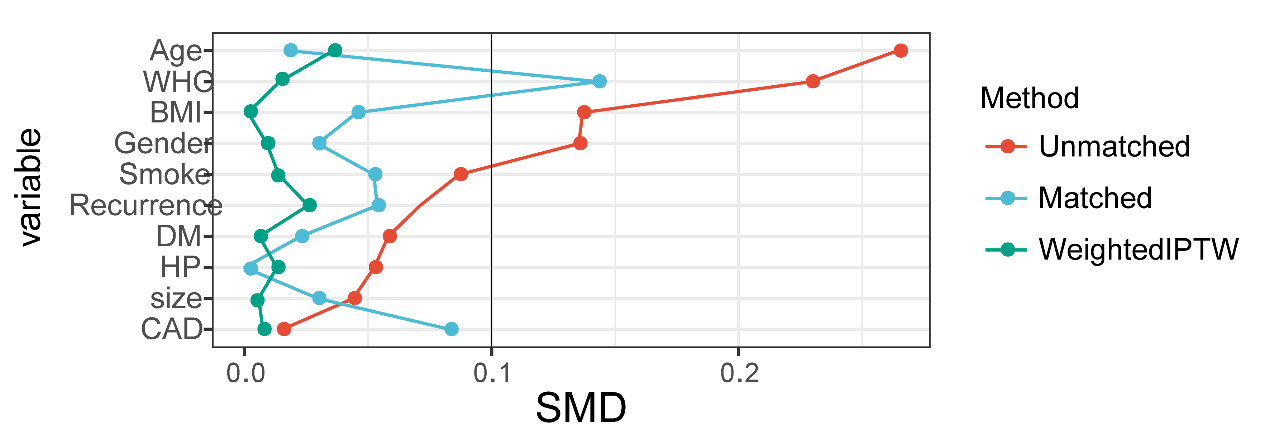


PSM analysis matched the above variables. The red line shows the SMD with crude analysis. The blue Line shows the SMD from a univariate logistic regression model with matching according to the propensity score. The green Line shows the SMD from a multivariable logistic regression model, with additional IPTW adjustment for the propensity score.
